# Understanding Hyaluronan Receptor (CD44) Interaction, HA-CD44 Activated Potential Targets in Cancer Therapeutics

**DOI:** 10.34172/apb.2021.050

**Published:** 2020-07-15

**Authors:** Gul-e-Saba Chaudhry, Abdah Akim, Muhammad Naveed Zafar, Naila Safdar, Yeong Yik Sung, Tengku Sifzizul Tengku Muhammad

**Affiliations:** ^1^Institute of Marine Biotechnology, Universiti Malaysia Terengganu, 21030 Kuala Terengganu, Malaysia.; ^2^Department of Biomedical Sciences, Universiti Putra Malaysia, Seri Kembangan, Selangor, Malaysia.; ^3^Department of Chemistry, Quaid-i-Azam University, Islamabad, 45320, Pakistan.; ^4^Department of Environmental Sciences, Fatima Jinnah University, Rawalpindi, Pakistan.

**Keywords:** Apoptosis, Cancer pathways, CD44, Cell proliferation, Hyaluronan, Targeted therapeutics, Tumor progression, Nanotechnology

## Abstract

Cancer is a complex mechanism involving a series of cellular events. The glycoproteins such as hyaluronan (HA) are a significant element of extracellular matrix (ECM), involve in the onset of cancer developmental process. The pivotal roles of HA in cancer progression depend on dysregulated expression in various cancer. HA, also gain attention due to consideration as a primary ligand of CD44 receptor. The CD44, complex transmembrane receptor protein, due to alternative splicing in the transcription process, various CD44 isoforms predominantly exist. The overexpression of distinct CD44 isoforms (CD44v) standard (CD44s) depends on the tumour type and stage. The receptor proteins, CD44 engage in a variety of biological processes, including cell growth, apoptosis, migration, and angiogenesis. HA-CD44 interaction trigger survival pathways that result in cell proliferation, invasion ultimately complex metastasis. The interaction and binding of ligand-receptor HA-CD44 regulate the downstream cytoskeleton pathways involve in cell survival or cell death. Thus, targeting HA, CD44 (variant and standard) isoform, and HA-CD44 binding consider as an attractive and useful approach towards cancer therapeutics. The use of various inhibitors of HA, hyaluronidases (HYALs), and utilizing targeted Nano-delivery of anticancer agents and antibodies against CD44, peptides gives promising results *in vitro* and *in vivo*. However, they are in clinical trials with favourable and unfavourable outcomes, which reflects the need for various modifications in targeting agents and a better understanding of potential targets in tumour progression pathways.

## ECM, a potential source of cancer progression initiators

**Figure 1:**
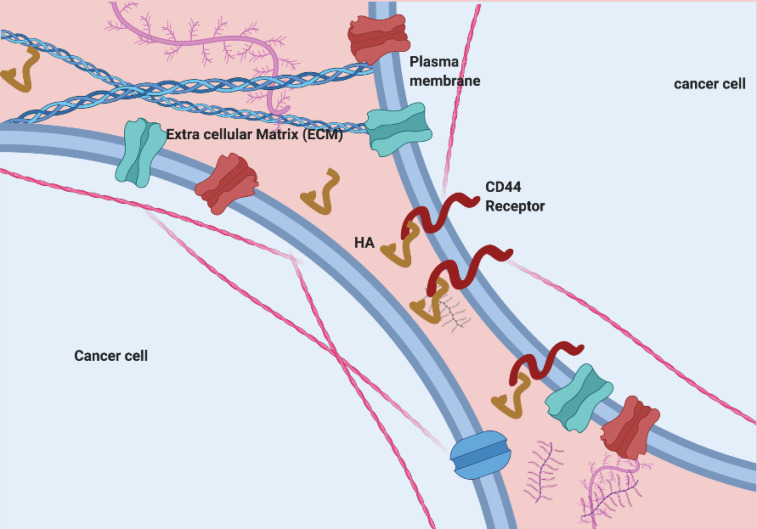
ECM, potential site for HA-CD44 communication

The extracellular environment is surrounding the cells considered as extracellular matrices (ECMs) necessary for support and communication between cells (Figure 1). The extracellular matrices composed of various glycol-proteins, which play an essential role in structure as well as functional aspects. The components include; i) proteoglycans, ii) proteins including collagens (fibrillar), iii) elastins proteins, iv) fibronectins proteins and v) laminins and the particular class of glycosaminoglycans (GAGs).^1,2^ These ECM components could act as ligand molecules, and due to ligand-receptor binding, the signal transduction pathways triggered that lead to a variety of disease propagation and cytoskeleton and chromatin structures organization aswell.^3-7^ Hyaluronan (HA) belongs to glycosaminoglycans, an integral hydrophilic part of the matrix. The surrounding proteins interact with integrin protein receptors where CD44 and RHAMM receptors got special attention due to cancer development and growth.^8,9^ The components in matrix regulate the fundamental process in the cell, any dysregulation considered as essential hallmarks of cancer.^10-17^ Additionally, ECM regulates the expression of stromal cells and indirectly the tumorigenic microenvironment. Any dysregulation leads to the development of inflammation and the generation of a disease microenvironment.^13^ The integrin-FAK signaling due to modification of the ECM results in reactivating dormant tumour cells.^18^ However, alteration in ECM is quite often due to variation in the expression level of matrix-degrading enzymes^19^. The alterations in ECM promotes the formation of a tumorigenic microenvironment leads to metastasis.^20^

## A major component of ECM, Hyaluronan

HA is linear repeating units of β glucuronic acid and β-N-acetyl glucosamine. HA, a principal component of the ECM in cancer cells, as well as normal cells, involved in various developmental process.^21^ Various enzymes involved in the synthesis of different molecular weight (MW) of HA. HA produced by HA synthases (HAS1-3). The disaccharides unit of N-acetyl-D-glucosamine and D-glucuronic acid polymerize via β-(1-3)-glucuronidic bond.^22^ Similarly, HA synthases HAS2 produces the larger HA more than 2 MDa; and HAS1 and HAS3 produces smaller HA with a range of less than 2 MDa.^23^ Along with synthases, hyaluronidases (HYAL1-3) fragment the higher MW HA to medium MMW (via HYAL2) and lower LMW (via HYAL1) into oHA.^24,25^ Alteration in a post-translational modification in the HAS2 produce mutations involve the replacement of one amino acid asparagines by another amino acid serines. Also, hyaluronidases (HYALs) mutations have linked to tumour resistance in the naked mole-rat.^26,27^

HA involve in various developmental processes such as cell growth, proliferation, migration, tissue development, embryogenesis, and wound healing.^28^ HA is the primary ligand of CD44 receptors.^30^ Various modifications in the splicing mechanism and glycosylation process produce of CD44 variants. These variants are differentiated by structural and functional aspects mainly responsible for a pro-inflammatory activity via cell-ECM component interacts.^22-25^ The HA-receptor mediated signal transduction occurs via CD44, the primary receptor for HA-binding and RHAMM (receptor for HA mediated mobility), toll-like receptors (TLR2, TLR4), HA receptor for endocytosis (HARE), HA-binding protein 1 (HABP1), and lymphatic vessel endothelial receptor for HA 1 (LYVE1).^28-32^

## CD44 receptor

**Figure 2:**
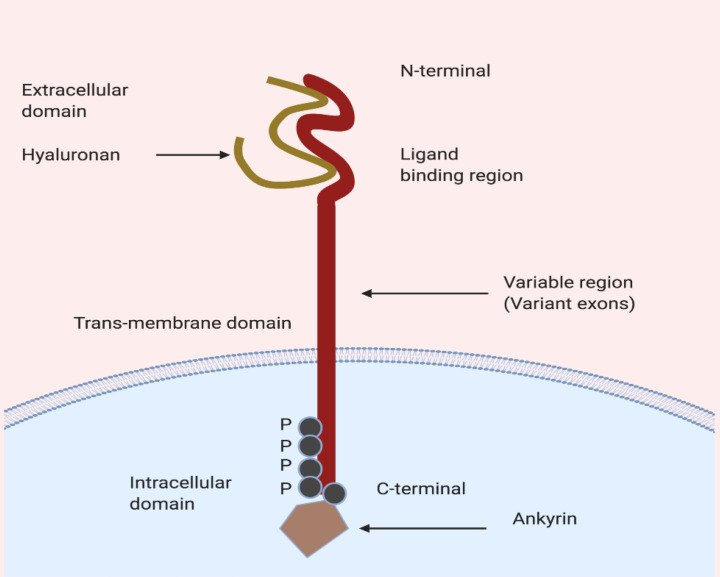
Schematic presentation of HA binding to cytoplasmic domain of CD44.

CD44 transmembrane protein, glycoprotein, encoded by 19 exons compromised gene positioned on chromosome 11 in humans.^33^ The protein is a transmembrane protein with three visible domains includes i) outermost extracellular domain, ii) middle transmembrane domain, and iii) innermost intracellular domain. The N-terminal region is essential for cellular interaction with ECM components such as ligand HA and due to the presence of area responsible for the formation of the various variant. During post-transcription modifications, alternative splicing occurs in variant exons (nine exons) results in the formation of variant isoforms (CD44v). However, constant exons encode the CD44 standard (CD44s). A-part from hyaluronic acid (HA), CD44 receptor have an affinity towards other ligands including osteopontin, collagen, chondroitin, fibronectin, and sulfated proteoglycan.^34-38^ The HA major ECM component is having a more binding affinity towards all isoforms of the CD44 receptor protein. The HA binds to CD44 (Figure 2) at extracellular domain causes conformational changes, which leads to activation of CD44 and recruitment of various other cytoplasmic and membrane-bound cytoplasmic proteins (adaptor molecules) trigger the downstream cell survival, growth and tumour progression pathways.^39^

## HA-CD44 interaction pathways in cancer progression

**Figure 3:**
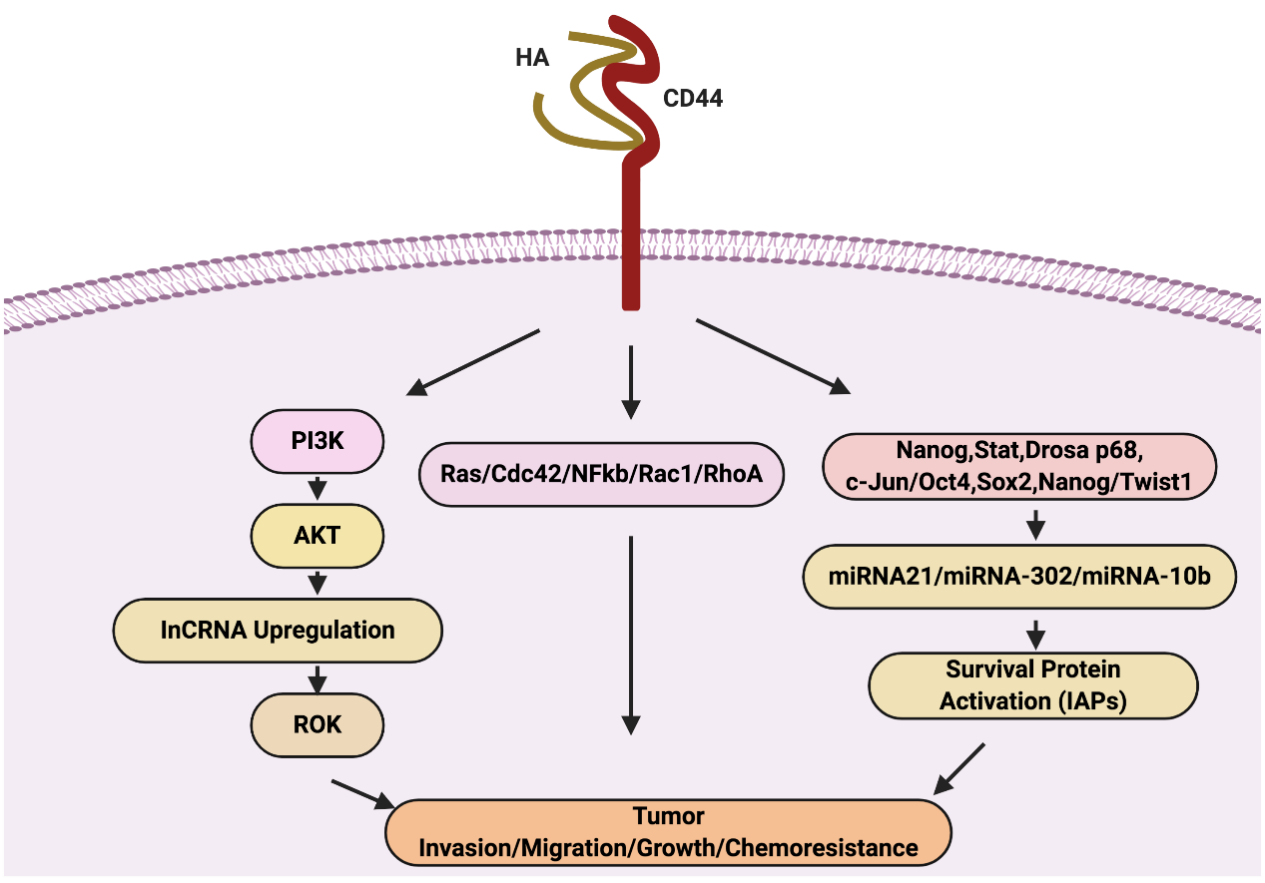
Therapeutic targets of tumor growth, invasion, metastasis and chemo resistance.

HA interaction with CD44 receptor leads to cell survival, cell growth, invasion, and metastasis via signaling networks include; RhoGTPases and PI3K/AKT pathway^40^ and other chemoresistance pathways via ROK activation (Figure 3). CD44 receptor triggers cell survival promoted the tumour metastasis. The HA ligand upon binding with CD44 triggers signal cascade via activating the cytoplasmic domain of CD44 and which results in the recruitment of various protein a series of cell signaling events. The CD44-cytoplasmic domain activated proteins such as ankyrin, merlin, and ERM involve in actin polymerization mediated by ERM proteins results in cytoskeleton rearrangements for tumour invasion and migration.^41^

CD44 interaction is associated with metastasis in several tumours.^42,43^ The role of CD44 in tumour progression quite clear; however, its involvement in trigger apoptosis inhibits tumour invasion and progression. CD44 in inducing apoptosis and reducing cell invasion is HA MW dependable but still contradictory which show the response differ in differ tissue also along with MW.^44,45^ The high molecular weight HA blocked invasion and small molecular weight oligomerized HA to initiate apoptosis and via inhibition the activation of cell survival and growth pathway (AKT) in breast, colon and mammary cancer cells.^46,47^

Besides AKT, RhoGTPases triggers a cascade of events from cell surface receptors to downstream signals. RhoGTPases acts as response molecules which act in the response of signal or external stimuli and regulates the various process of cytoskeleton reorganization results in cell invasion and metastasis. RhoGTPases belong to Ras superfamily, includes RhoA, Rac1, and Cdc42. These Rho proteins exist in an inactive form and convert to active form upon binding of GTP molecules. The RhoGTPase involve in normal cell invasion and development. However, the overexpression in various human cancers link them to cancer progression and poor prognosis.^48-51^ Additionally, an important kinase as Rho kinase (ROK) regulates the process of tumour invasion and metastasis via myosin light chain phosphatase. The myosin light chain phosphatase promotes the secretion of ECM degrading proteins as matrix metalloproteins (MMPs). The activation of MMPs via CD44 receptor protein help in invasion and also angiogenesis via degradation of collagen.^52,53^ The MMPs include MMP-2 (type IV collagenase), MMP-9 (type V collagenase), MMP-3 (stromelysins) and MMP-1 (interstitial fibroblast-type collagenase) degrade ECM materials, crucial in tumor invasion and metastases.

HA/CD44 interaction mediates cytoskeleton activation via critical RhoA signaling. RhoA regulation cellular functions via alteration of intracellular Ca^2^ levels. The intracellular Ca^2^ mobilization mediated via essential phospholipase C (PLC). Upon activated, PLCs converted to inositol trisphosphate (IP3) via PIP2 (hydrolyzed), which results in the release of Ca^2^ from deposit storage. This released Ca^2^ stimulates cytoskeleton rearrangement and eventually tumour progression.^54-56^ In breast tumour cells107-109, HA activation of CD44 leads to the expression of multidrug resistance gene (P-glycoprotein) and anti-apoptotic gene Bcl expression, which promotes tumour cell proliferation and survival.^57-59^ Apoptosis is a highly regulated mechanism of cell death characterized by morphological and biochemical changes, including various enzymes proteins that lead to cell death and maintain cell growth.^60^

However, the recent proposed HA-CD44 induced tumour progression signaling events in cancer by Bourguignon 2019.^61^ The interaction and binding of HA to CD44v upregulate Ras/Cdc42/Rac1/RhoA/NFkB expression, and various cytokines production involve in tumour cell survival, growth, invasion, and migration. As we already discussed, HA-AKT activation stimulates PI3K/AKT activation also the production of LncRNA (UCA1). LncRNA triggered tumour cell migration and invasion via ROK. Moreover, HA/CD44 also responsible for chemoresistance via activation of transcriptional factor-induced miR-10b, miRNA-21, and miR-302 expression. The JNK activity causes activation of c-Jun via phosphorylation. The activated c-Jun binds to the miR-21 promoter and induces the up-regulation of miR-21 expression. Similarly, HA-CD44 interaction triggers the complex formation include; Nanog, Stat-3, DROSHA, and p68. This complex bind to the miR-21 promoter region, resulting in the expression of miR-21. The miRNA expression upregulates IAP protein, involved in tumour cell survival, and chemoresistance. Activated miR-21 stimulates upregulation in RhoC and ROK-regulated tumour cell migration and invasion. In contrast, activated miRNA-302 expression involves in chemoresistance also due to HA-CD44 interaction. HA binding to CD44 promotes the recruitment and build association between cytoplasmic domain of CD44v3 and OCT4/SOX2/Nanog. This OCT4/SOX2/Nanog induce IAP (cIAP-1, cIAP-2, and XIAP) expression via the upregulation of miR-302 gene expression. IAPs involve in tumour cell growth and chemoresistance in tumour cells. Moreover, the cytoskeleton activation by miRNA-10b expression trigger via HA-CD44 interaction. HA binding to CD44 promotes c-Src activation via phosphorylation, the activated c-Src causes phosphorylation of Twist. The activated Twist acts as TF, activate the mR-10b expression, promotes tumour cell migration, and invasion.

## HA and CD44 role in Cancer regulation

The microenvironment outside the cell is a critical regulator of the fundamental growth of the cell. Alteration in cell growth trigger rapid growth and migration causes metastasis.^62^ HA is biopolymer and the primary component of ECM, provides a hydrophilic part to the matrix and cellular support but gain special attention when it comes to cell survival, growth, and differentiation.^63^ However, HA contributes to abrupt growth and invasion of cancer cells. Due to its hydrophilic nature, HA can form a protective lining around the tumour cell to protect the tumour cells from immune attack.^64,65^ Any transcriptional or translational disruption in HA synthases or hyaluronidases can lead to over or fragmented production of HA. In several tumours, HAS and HYAL regulate the production of HA, which linked to tumour development and metastasis. Besides, HYAL fragmented HA some ROS-fragmented HA also found to be involved in overproduction of HA in tumours growth, invasion and spreading.^66^ The over-expression of synthase HAS2 and HAS3 results in enhancing HA overproduction, which triggers the production sarcomas and other melanoma cells.^67,68^ HA trigger the formation of new blood vessels and also circulation of tumour cells via growth and invasion seen in some studies. HA induce angiogenesis and lymphatic invasion in tumour transgenic mouse models.^69,70^ Apart from the above concern, interstitial fluid pressure cause by HA also highlighted in drug delivery. By using HYALs, it degrades HA, reduces the pressure, and improve the efficacy of therapeutics.^71^

The CD44 exists in various isoforms and gain much importance due to involvement in the pathogenesis of cancer. The variant form of CD44v could use as a biomarker for disease (cancer) prognosis. Several isoforms linked to specific cancer and play a role in cell growth and metastasis. The CD44v found to be involved in tumour progression and invasion. The overexpressed form of CD44v6 linked to invasion in endocrine-resistant breast cancer cells via activation of epidermal growth factor receptor signalling.^72^ As cancer is a multistage disease, and the CD44v expression also have an association with various stages which ensure the precise involvement of CD44v in tumour progression. CD44v6v appearance only noticed in advanced stages of pancreatic cancer during the metastatic behaviour of cells and not visible in a benign tumour.^73^ Increased expression of CD44v6 in pancreatic cancer link to metastasis.^74,75^ The CD44v6 role in metastasis confirmed by transfection study confirms the involvement of CD44v6 in rat pancreatic adenocarcinoma.^76^ Various treatment against CD44v6 linked metastasis include bFGF, EGF, TNFα, and TGF-β1(growth factors and cytokines) unable to reduce the surface expression of CD44v6 receptor. Similarly, interferon (IFN) γ also not wholly inhibit the expression of CD44v6.^77^ The studies also confirm the CD44v as a survival marker. The presence of enhancing expression of CD44v6 link to shorter survival time as compared to tumours deprived of CD44v6.^78^ Also, the positive expression of CD44v6 and CD44v9 together link to metastasis with low survival rate.^79,80^ Also, the standard form of CD44 regulates the metastasis in tumour cells. Studies show that down-regulation of CD44s detected in human metastasis tissue.

Silencing the expression of CD44v9 (via short hairpin RNA) overturned the gathering of various proteins (HSP90, PI3K, and others) into lipid raft-like structures and attenuate the downstream signal cascade of tumour progression.^81^ The isoforms CD44v6 and CD44v9 overexpressed in prostate cancer (PC3-epithelial cells).^82^ The function of CD44v6 evaluated using small interfering RNA (siRNA) in prostate cancer cells and Knock-down of CD44v6 linked to increasing chemo/radiosensitivity.^83^ Similarly, overexpression of CD44v3 isoform triggered a substantial increase in cell migration. In neck squamous cell carcinoma (HNSCC), elevated CD44v3 levels noticed.^72^ The inhibitor study using, anti- CD44v3 antibody shows the reverse cisplatin resistance and decrease cell proliferation.^84^ In colorectal cancer, CD44v6 and CD44v10 overexpression detected which linked to poor prognosis, cell migration and metastasis.^85-87^ Various cytokines regulate CD44v6 expression, which activates Wnt/β-catenin pathway and promotes migration and metastasis.^88^ Previous study shows that CD44v operates in a specific and overlapping manner and by studying CD44v help to understand the various variant as a potential target in drug delivery. The variant form CD44v8-10 involve in the protection of gastrointestinal cancer cells against ROS (reactive oxygen species), CD44v4-10 and CD44v8-10 promoted adenoma and gastric tumour initiation.^89,90^ Similarly, another variant CD44v9 found to be overexpression gastric adenocarcinoma.^91^ Overexpression of CD44v6 activate the phosphorylation of AKT, autophagy flux, and ERKs which result in cell survival and growth. The colon cancer cells treated with chemotherapy drugs with enhancing the expression of CD44v6 reduce cytotoxicity.^92^

## Strategies to target HA and CD44 interaction

### HA as a therapeutic target

The rich level HA in various tumours and metastatic cancers responsible for cell growth proliferation, invasion, angiogenesis, metastasis along with acquiring resistance in response to anticancer agents. HA abundance in pancreatic ductal adenocarcinoma links to a lower survival rate. HA interaction with CD44 results in cell growth, invasion, and metastasis. To prevent the interaction and downstream pathways of cancer propagation, HA ligand, CD44 receptor with its variants, and HA-CD44 interaction could consider as important targets in drug development. In the case of HA, the strategies are i) hyaluronidases along with chemotherapeutics, ii) use of HA oligosaccharides (oHA) to induce apoptosis, iii) inhibitors of HA and iv) HA-base drugs.

HYALs are well-known enzymes that can fragment HA. Previous studies demonstrate that the expression of wild type HYAL-1 (HYAL-1-v1) induces apoptosis via G2-M arrest in bladder cancer cells.^60^ However, due to adverse impact as an anti-adhesive on normal tissue, HYALs have limitations and side effects.^93^ Interestingly, commercial bovine testicular or bacterial HYAL act as an anti-adhesive compound on EMT-6 tumour spheroids improve the chemosensitivity by increasing the accessibility.^94^ In regards to drug therapeutics, HA also increase the intestinal fluid pressure and hinder the drug penetration in pancreatic ductal adenocarcinomas. The application of PEGPH20 increases vessel size and recover the increased interstitial fluid. The treatment of Gem+PEGPH20 in placebo control trials in KPC improves the survival rate and significant reduction of metastatic tumour burden.^95^ Interestingly, HA oligosaccharides (oHA) trigger the induction of apoptosis in lymphoma cell lines via inhibition of downstream cancer survival pathways. The oligosaccharides (oHA) inhibited PIP3 production, involve in PI3-K activation and results in deactivation of Akt (via reduced phosphorylation) without involving NF-kB activation in lymphoma cells.

HA found to be abundant in most cancers. Previous studies show that 4-methylumbelliferone (MU) inhibitor of HA synthesis, deplete uridine diphosphate glucuronic acid, and the down-regulation of HAS human skin fibroblasts. Similarly, MU inhibits HA production in pancreatic cancer cells (KP1-NL) and decreases liver metastases in mice injected with pancreatic cancer cells.^96^ 4-Methylumbelliferone was then later shown to inhibit HA production and to have anticancer activities in mouse models of various cancers. However, the inhibition depends on MW of HA, as HMW-HA revoke the inhibitory and non-metastatic effects of MU on MIA PaCa-2 cells. Studies have shown that MU inhibits HA synthesis in at least two ways. First, MU depletes UDP-GlcUA, which is a precursor of HA.^97^ Second, MU reduces the expression of HAS mRNA.^98^ However, no studies have described the mechanisms through which MU reduces HAS mRNA expression. However, more studies need to do as the MU in inhibition of overall HAS mRNA and effect on CD44 expression still not clear yet. Some reports have indicated that MU suppresses HAS mRNA expression,^99-101^ whereas another report stated that HAS mRNA expression upregulated.^102^ However, its right to be said that the effect of MU on expression depends on various cell types with specific up-regulation or down-regulation.

HA, a versatile biopolymer, increases the solubility of poorly soluble anticancer drugs. HA improve the efficacy of anticancer drugs with improving solubility and biocompatibility via various mode as follows; i) HA-conjugate drugs, ii) HA-encapsulated drugs, iii) gagomers, iv) micelles and v) Nanocarriers. The paclitaxel (PTX), the potent antimitotic agent inhibits cell growth, but due to poor water solubility limits its application in drug therapeutics. The HA-PTX conjugated drug name as HYTAD1-p20 with improve solubility and biocompatibility in the bladder and other cancers.^103,104^ Similarly, HA-conjugated-PTX-N-hydroxysuccinimide ester (PTX-NHS) and HA-SN-38 (CPT11 (irinotecan) metabolite) used against CD44 expressing cell line (colon, breast, gastric and others) reduced tumour cell growth and metastasis.^105-109^

The side effects of potential anticancer also hinder the application of potential compounds in drug delivery. However, *in-vitro* and *in-vivo* HA encapsulated drugs, HA-5-fluorouracil (HyFIVETM) and HA-doxorubicin (5- FU) show reasonable cytotoxic efficacy, undergoing clinical trials.^110,111^ Self-assemble Lipid molecule coated with hyaluronan (HMW) as liposomes or lipoplexes loaded drug deliver plasmid DNA and siRNA (small interfering RNA) also used against several cancers including head and cancer, expressed CD44 receptor.^112-117^ The HA micelles are self-organized structure, HA polymer (carboxyl group) conjugate to another polymer, i.e., PEG (polyethylene) amino group. HA-micelles with paclitaxel (HA-PTX), doxorubicin (HA-DOX) HA-DOX, and HA-salinomycin produced effective cytotoxicity on DC44 expressing cells.^118-120^ Nanoparticles act as Nanocarriers for delivery the therapeutic agent to target site hold promising features include; dots (quantum and nanodots, nanodots graphene,^121-123^ carbon nanotubes,^124^ and nanoparticles gold and iron oxide nanoparticles.^125,126^ and silica nanoparticles,^127^ and they have found to acquire novel characteristics after their conjugation with HA.^128^ Due to improve the efficacy of HA conjugated nanocarriers delivered anticancer drugs such as HA-epirubicin,^129^ HA-DOX,^115^ HA-PTX,^130,131^ and HA-MMC,^129^ as well as HA-siRNA make HA attractive polymer in drug delivery. Similarly, drug-resistant tumor cell treatment improved via combination therapy of HA (HA–CUR/DOX-NPs). The improve efficacy was achieved via down-regulate the expression of P-gp and induce apoptosis (regulating Bax/Bcl-2),^132^ thus increasing the therapeutic effect.

### CD44 as a therapeutic target

The CD44 essential receptor protein involves in cancer cell growth, survival, and metastasis in the majority of cancer. HA is the primary ligand of CD44 receptor; upon binding, to the receptor, the ligand-receptor interaction activates the down-stream cancer pathways.^133^ CD44 do exist in various isoforms of CD44v along with CD44s play a significant role progression of cancer and prognosis.^134-136^

Several studies regarding the role of CD44s and CD44v in the onset of cancer propagation. However, the function of CD44 isoforms differs in various cancers and different outcomes CD44 isoforms. As in one study, the CD44s (standard isoform) aberrant expression or loss is linked to an increased risk of metastasis, as observed in malignant breast cancer.^135^ Along with this, CD44s indorsed invasion in breast tumour and liver metastasis.^136^ Amongst isoforms of CD44, CD44v (3, 6 and, 7 and 8) linked to the propagation of breast cancer.^137^ In lymph node metastasis, CD44v6 expression was upregulated, while both CD44v (v3 and v6) were upregulated invasive cribriform breast tumours. However, the downregulation in CD44v4 in the invasive cribriform breast but upregulated in trans-endothelial cell metastasis. More research needs to done further to clarify the specific role of CD44s and CD44v variants.

Antibodies to inhibit the CD44 mediated cancer progression also show remarkable results in inhibition of HA-CD44 downstream pathways. The binding of antibodies (anti-CD44) to CD44 variants that expressed in a variety of cancers interfere with the binding of HA to CD44 receptor cells and disrupt CD44 matrix interactions. This interruption cause alteration in CD44, triggered cancer progression pathways as well as induce apoptosis.^138^ The use of several peptides, The Pep-1 and BH-P, inhibit the proliferation of melanoma tumour cells in nude mice xenograft models.^139^ The CD44v6 isoforms identified as markers of CSCs in colon cancer. As a receptor CD44v6 involve in matrix assembly and link with metastasis.^140,141^ Therefore, targeting CD44v6 in colon cancer is a promising therapeutic approach. The siRNA/shRNA use in the inhibition of CD44 receptor expression successfully blocks the interaction (HA-CD44) and induction of cancer progression pathways. The using cell-specific shRNA delivery approach to inhibit HA-CD44v6 interaction inhibited distant growth in colon tumour and downstream tumour progression pathways.^142,143^

The double approach in targeting CD44 by using an antibody as well as cytotoxic drugs have studied. The cytotoxic drug (mertansine) conjugated to an antibody against CD44v6 used in early phase clinical trials.^144,145^ Similarly, another drug bivatuzumab (anti-CD44v6 monoclonal antibody) along with combinational drug mertansine also undergoing clinical trials.^146,147^ However, due to toxicity towards healthy cells (CD44v6 positive expression), the phase I clinical trials did not consider as successful. By studying this limitation of antibody therapy, the quest for alternative therapies increased.

Natural products show the remarkable result in cancer therapeutics. Besides various synthetic inhibitor, different natural potential compounds such as silibinin, zerumbone, gemini, curcumin, epigallocatechin gallate, and apigenin down-regulate the expression of CD44 isoforms.^148^ Silibinin bioactive component, isolated from plant *Silybum marianum* used in phase II clinical trials in prostate cancer and. Silibinin inhibits cell growth in a time-dependent manner in BxPC3 and Panc1 pancreatic cancer cell lines. The active compound (silibinin) acts as a transcription regulator in CD44 expression decreased the expression of levels of CD44v7-10 RNA and well as translated protein in prostate cancer cells.^149-151^ Another potential regulator of the CD44 receptor cell, Zerumbone, belongs to the terpene class of phytochemicals. The expression level of CD44 in breast cancer cells downregulated by Zerumbone via inhibiting phosphorylation of STAT3 which involve in EGF-induced CD44 expression.^152-154^ Similarly, members of vitamin D family Gemini also act as a transcription regulator. As a transcription regulator, it downregulates the expression of CD44 protein level, inhibited the invasion and metastasis in mice model.^155,156^ The enhance synergistic effect of curcumin (potential active compound) and epigallocatechin gallate (catechin) inhibit the expression of the CD44 receptor on cancer cells. The combine approaches successfully reduced CD44- positive (CSC) and downregulate the phosphorylation of STAT3 expression.^157^ Also, potent phytochemical apigenin belongs to flavonoid group improve the cell survival in a prostate cancer patient by dose-dependent inhibition in expression of CD44 in CSC by upregulating the expression of p21 and p27.^158^ Along with previous reports and our research various natural extract and fractions rich of potential phytochemicals and targeted delivery produce effective cytotoxicity via induction of apoptosis (*in-vitro*).^159-167^ However, the study of receptor-ligand molecular interactions required to understand the inhibition of anti-apoptosis pathways involves in cancer progression.

## Conclusion

HA versatile biopolymer, the primary ligand of CD44 receptor cells. HA-CD44 interaction and binding can regulate cytoskeleton protein activation in various tumour cells. The cytoskeleton activation triggers different downstream pathways involved in tumour propagation. The HA and CD44, along with signaling proteins in the cytoskeleton could be suitable markers for cancer drug therapeutics. The CD44 exists in various isoforms and regulate the cell survival signaling pathways in a variety of cancers. The alternative splicing triggers the variant isoform of CD44, and the expression level is a critical regulator in various cancers. Here, we discussed the understanding of the HA-CD44 interaction/binding and discussed different activated pathways involve in cancer cell growth, survival, and metastasis via signaling networks include; RhoGTPases and PI3K/AKT pathway. HA-CD44 activation stimulates AKT activation via ROK (kinase) triggered tumour cell migration and invasion. Also, HA/CD44 also responsible for chemoresistance via the up-regulation of miR-10b, miRNA-21, and miR-302 expression. The overexpression of various CD44 isoforms depends on different cell types and stages—the fate of cancer cells regulated by isoforms, CD44v, and standard CD44s. Thus, targeting HA by using HYALs, targeting HA synthase and HA-inhibitors, useful strategy to inhibit HA trigger CD44-signaling pathways. Similarly, targeting CD44(variant) by application of CD44 anti-bodies, peptides HA-Nano carries and natural CD44 negative regulator showing optimal success. Thus, extensive studies show that CD44 has the potential to be an effective drug therapeutics.

## Ethical Issue

Not applicable.

## Conflict of Interest

Authors have no conflict of interest.

## Acknowledgments

The authors would like to acknowledge the postdoctoral fellowship provided to Dr Gul-e-Saba Chaudhry. The Figures were created by using Biorender.
